# Non-invasive continuous cardiac output monitoring in thoracic cancer surgery: A comparative study between calibrated pulse contour analysis and chest bioreactance

**DOI:** 10.1097/EA9.0000000000000006

**Published:** 2022-09-15

**Authors:** Jean-Luc Fellahi, Paul Abraham, Nicolas Tiberghien, Clément Coelembier, Jean-Michel Maury, Karim Bendjelid

**Affiliations:** From the Service d’Anesthésie-Réanimation, Hôpital Louis Pradel, Hospices Civils de Lyon (J-LF, NT, CC), Laboratoire CarMeN, Inserm UMR 1060, Université Claude Bernard Lyon 1, Lyon, France (J-LF), Service de Médecine Intensive Adulte, Centre Hospitalier Universitaire du Vaud, Lausanne, Switzerland (PA), Service de Chirurgie Thoracique, Hôpital Louis Pradel, Hospices Civils de Lyon, Lyon, France (J-MM), Département d’Anesthésiologie et de Soins Intensifs, Hôpitaux Universitaires de Genève, Genève, Switzerland (KB)

## Abstract

**BACKGROUND:**

Patients scheduled for thoracic cancer surgery are eligible for goal-directed fluid therapy, but cardiac output monitoring remains challenging in that specific setting.

**OBJECTIVE:**

We aimed to compare cardiac output as measured with chest bioreactance with that measured by calibrated pulse contour analysis; the hypothesis being that both methods would be interchangeable.

**DESIGN:**

A prospective monocentre observational study.

**SETTING:**

A tertiary university hospital.

**PATIENTS:**

Fifty adult patients undergoing thoracic cancer surgery over a one year period.

**MAIN OUTCOME MEASURES:**

Simultaneous measurements of cardiac index (CI) with bioreactance (CI-NICOM) and arterial pulse contour analysis calibrated by transthoracic echocardiography (CI-PCA) were performed at eight pre-specified intra-operative time points and following fluid challenge and/or vasoactive agents. Relationships between absolute values and changes in CI were assessed by linear regression. Interchangeability was tested with Bland–Altman analysis and percentage error calculation. A four quadrant plot was used to evaluate trending ability.

**RESULTS:**

There was a significant difference between CI-PCA and CI-NICOM: 2.4 ± 0.8 (range: 0.9 to 5.8) l min^−1^ m^−2^ vs. 2.9 ± 0.9 (range: 0.9 to 7.2) l min^−1^ m^−2^, respectively (*P* < 0.001). A positive relationship was found between both techniques: y = 0.29x + 2.19; *r*^2^ = 0.08 (*P* < 0.001). Taking CI-PCA as the reference method, there was a systematic overestimation of CI-NICOM by 21% (0.5 l min^−1^ m^−2^) and limits of agreement were large: -2.49 to 1.47 l min^−1^ m^−2^. The percentage error was 77% and concordance rates were 75 and 70% with and without an exclusion zone of 0.5 l min^−1^ m^−2^.

**CONCLUSION:**

Chest bioreactance is feasible and well tolerated in patients undergoing thoracic surgery for cancer. When compared with calibrated PCA over a wide range of CI values, the technique is moderately correlated, not interchangeable, and provides moderate trending ability.

**TRIAL REGISTRATION:**

NCT04251637.

**KEY POINTS**

- Non-invasive cardiac output monitoring with chest bioreactance is well tolerated, easily feasible and able to detect intra-operative spontaneous changes in patients undergoing thoracic surgery for cancer.
- Non-invasive cardiac output monitoring with chest bioreactance is moderately correlated, provides moderate trending ability but is not interchangeable with calibrated pulse contour analysis over a wide range of CI values.

## Introduction

Peri-operative individualised goal-directed fluid therapy (GDFT) has been found to improve patient outcomes.^1–3^ However, its implementation remains limited in routine practice.^4^ Inadequate volume therapy can result in deleterious effects, especially in frail or high-risk patients, so fluid administration should be monitored and titrated using measurements of stroke volume and/or cardiac output.^5^ However, less than one-third of high-risk patients actually have cardiac output monitored during the peri-operative period, mainly because reference methods are considered as too invasive, too time-consuming in the operating room and too complicated for daily use at the bedside.^6^ Non-invasive, automated, plug-and-play systems have been developed over the past 20 years. Even though they are less accurate and not interchangeable with reference methods,^7^ they could be useful to assist decision-making processes and improve outcomes.^8,9^ Chest bioreactance (NICOM) is one of those easy-to-use technologies enabling non-invasive, continuous, operator-independent measurement of cardiac output.^10,11^ Following cardiac surgery, bioreactance has been shown to be comparable with pulse contour analysis (PCA) calibrated by transpulmonary thermodilution and it performed similarly to oesophageal Doppler in guiding GDFT in colorectal surgery patients.^12,13^

Patients scheduled for thoracic cancer surgery are most often considered as intermediate-to-high risk surgical patients and are eligible to GDFT, as part of a global ERAS (Enhanced Recovery after Surgery) programme. Such an individualised fluid strategy should replace the traditional ‘keep the lungs dry’ with its potential for peri-operative hypoperfusion and postoperative complications in some patients. Both PCA and oesophageal Doppler have been proposed for monitoring cardiac output in thoracic surgery with contrasting results.^14,15^ The former necessitates the use of an invasive arterial pressure catheter, which is not always accepted as a standard of care by clinicians, while the latter can be seen as not easy-to-use and operator-dependent. Thoracic surgery is challenging for cardiac output monitoring technologies because it requires both supine and lateral positioning, alternating two-lung and one-lung ventilation, and closed and open-chest conditions. To date, NICOM has not been assessed in the specific setting of surgical patients who undergo several ventilation manoeuvres and postures that affect haemodynamic load conditions.

Therefore, using calibrated PCA as the reference method, we aimed to assess the feasibility, safety and reliability of NICOM in thoracic cancer surgery. We tested the hypothesis that NICOM would show a good trending ability with calibrated PCA and could thus be used interchangeably.

## Materials and methods

A monocentre observational prospective study was conducted over a one year period (from 1 March 2020 to 13 March 2021) at the Teaching University Louis Pradel Hospital (Lyon, France). The study protocol was registered with ClinicalTrials.gov (ID: NCT04251637). The study was in accordance with the STARD Statement concerning diagnostic accuracy studies (Annex 1).^16^ Only adult patients (> 18 years) who were scheduled for elective unilateral video-assisted or open-chest thoracic cancer surgery under general anaesthesia with PCA cardiac output monitoring via an indwelling radial artery catheter were included in the study. Patients excluded comprised pregnant women, patients younger than 18 years, patients unable to give consent or who required legal protection measures, patients undergoing emergency surgery or in whom invasive PCA cardiac output monitoring was not planned, and patients with poor quality echocardiographic imaging.

### Ethics

Ethics approval for this study (CPP Ref#19064–36153) was provided by the Research Ethics Committee of Ile de France XI, 20 rue Armagis, 78105 Saint-Germain-en-Laye Cedex, France (Chairperson Prof. Sabine de la Porte) on 27 September 2019. According to the French law and because data were being collected during routine care that conformed to standard procedures currently used in the institution, authorisation was granted to waive written informed consent. Verbal consent was, however, obtained from all participants before surgery.

### Peri-operative management

General anaesthesia and peri-operative management followed institutional standards and were similar in all patients who were *a priori* included into an ERAS programme. Briefly, intra-operative monitoring techniques included continuous five-lead electrocardiogram with computerised analysis of repolarisation, pulse oximetry, continuous invasive arterial blood pressure by means of a radial artery catheter and depth of anaesthesia by means of the bispectral index. Forced-air warming was also used in all patients. Target controlled total intravenous anaesthesia (effect site concentrations of propofol and either remifentanil or sufentanil) or inhaled sevoflurane in order to maintain a BIS value between 40 and 60 were used at the discretion of the attending anaesthesiologist. After induction of anaesthesia, muscle relaxation was obtained with rocuronium and all patients were intubated with a double-lumen tube, whose position was visually checked by means of bronchial endoscopy. A nasogastric tube was routinely inserted. Mechanical ventilation was in accordance with our standard of care: a positive end-expiratory pressure of 5 cmH_2_O, tidal volume 6 to 8 ml kg^−1^ predicted body weight (PBW) during two-lung ventilation and 4 to 5 ml kg^−1^ PBW during one-lung ventilation: the respiratory rate was left to the discretion of the anaesthesiologist, but was usually increased to maintain end tidal CO_2_. The use of thoracic epidural analgesia, thoracic paravertebral block or single shot serratus plane block in addition to the general anaesthesia was at the discretion of attending anaesthetists. Surgical techniques included video-assisted or open-chest surgery according to international guidelines. A single chest tube was placed at the end of surgery and extubation was performed in the operating theatre. All patients were admitted to the post-anaesthesia care unit for at least 2 h and then carefully assessed before transfer to the ward.

The radial artery catheter (ProAQT; Pulsion-Getinge, Feldkirchen, Germany) was connected to the stand-alone PulsioFlex™ monitor (Pulsion-Getinge). Continuous PCA cardiac output measurement was initiated after an initial external calibration of the system by a triplicate transthoracic echocardiography cardiac output calculation as follows: aortic VTI (cm) x aortic section area (cm^2^) × heart rate (beats min^−1^). Subsequently, fluid challenges and/or boluses of intravenous ephedrine or norepinephrine were given intra-operatively, as appropriate, to maintain mean arterial pressure above 65 mmHg and cardiac index (CI) more than 2.0 l min^−1^ m^−2^. After cleaning the skin by rubbing with an alcohol swab, four bioreactance electrodes (Starling sensors CMS25, Cheetah Medical Ltd, Israel) were carefully applied to the skin at the four corners of the thoracic body surface and connected to the Starling™ monitor (Starling Monitor CMMST5S, Cheetah Medical Ltd, Israel) to obtain a NICOM signal. Following a 3 min period of auto-calibration, cardiac output and stroke volume were continuously recorded and stored on-line for further analysis. When compared with older systems of thoracic bioimpedance, the NICOM signal is based on the frequency-modulation and phase-modulation of the output voltage. Compared with bioimpedance, bioreactance technology yields a signal to noise ratio improvement of some 100-fold.^10^

### Study protocol

The protocol was initially designed to compare bioreactance with both calibrated PCA and oesophageal Doppler (ClinicalTrials.gov; ID: NCT04251637). However, after the first 10 patients, it became clear that oesophageal Doppler was unsuitable for monitoring cardiac output in our specific surgical setting, so we abandoned that arm of the study.

Eight pairs of simultaneous measurements of CI and stroke volume index (SVI) by calibrated PCA (CI-PCA and SVI-PCA) and chest bioreactance (CI-NICOM and SVI-NICOM) were recorded at pre specified intra-operative time points for each patient: T0 = post induction of general anaesthesia, two-lung ventilation, closed chest, supine position; T1 = two-lung ventilation, closed chest, lateral positioning; T2 = one-lung ventilation, closed chest, lateral positioning; T3 = one-lung ventilation, open-chest, lateral positioning; T4 = washing of pleural cavity with 1000 ml of 0.9% saline; T5 = before lung recruitment manoeuvre; T6 = during a standardised lung recruitment manoeuvre (positive end-expiratory pressure 30 cmH_2_O for 30 s); T7 = end of surgery. When fluid challenge and/or vasoactive agent administration were ordered by the attending anaesthesiologist, additional intra-operative time points were recorded.

### Endpoints

The primary endpoint of the study was the interchangeability of CI-PCA and CI-NICOM as assessed by Bland–Altman analysis and percentage error calculation. As previously described, a percentage error less than 30% would mean that both techniques are interchangeable.^17^ Secondary endpoints were feasibility and safety of chest bioreactance monitoring in thoracic cancer surgery; relationships between absolute values and maximum changes of CI-PCA and CI-NICOM; and trending ability of NICOM when compared with calibrated PCA.

### Statistical analysis

Considering preliminary results previously obtained in 10 thoracic surgical patients, we calculated that 370 paired data points would be necessary to show a mean bias of 0.25 l min^−1^ m^−2^ with a standard deviation of bias of 0.60 l min^−1^ m^−2^ and a maximal difference of 1.60 l min^−1^ m^−2^ (α risk = 0.05 and β risk = 0.10). Thus, considering potential missing data, we decided to include 50 patients in order to obtain 400 paired data points.

Data are expressed as mean ± SD or median [IQR] for non-normally distributed variables (Kolmogorov–Smirnov test) or number (%), as appropriate. Categorical variables were compared with a χ^2^ test and continuous variables were compared with a *t*-test or a Mann–Whitney *U* test, depending on their distribution. Correlations between absolute values of CI-PCA and CI-NICOM and between maximum changes in CI were determined by linear regression and heat map. Comparison between correlation coefficients was made by a *Z*-test. The Bland–Altman analysis was used to compare the mean bias and limits of agreement of both CI-PCA and CI-NICOM measurements. Because multiple measurements were performed in the same individuals, the classic analysis was replaced by a technique dedicated to the evaluation of the agreement between methods of measurement with multiple observations per individual.^18^ Percentage error was calculated as (1.96 × SD)/CI, where SD was standard deviation of bias and CI was the mean value of CI-PCA used as the reference technique.^17^ Trending analysis was performed with four-quadrant plots and the concordance rate was calculated with and without application of an exclusion zone set as 0.5 l min^−1^ m^−2^, a value that has been previously considered as optimal.^19^ A concordance rate of more than 90% to 95% indicates reliable trending ability.^19^

A *P* value of less than 0.05 was considered as statistically significant and all *P* values were two-tailed. Statistical analyses were performed using MedCalc® Statistical Software version 20.023 (MedCalc Software Ltd, Ostend, Belgium; https://www.medcalc.org; 2021).

## Results

Fifty patients were included over the study period and data from 465 CI paired data points were collected. Patient and pre-operative and intra-operative clinical characteristics of the whole cohort of patients are reported in Table 1. Video-assisted thoracic surgery was used in 62% of cases and the vast majority of patients were at intermediate risk, as shown by median values of ASA and surgical Apgar scores^21^ (Table 1). Intra-operative bleeding was low and the total fluid balance at the end of surgery was slightly positive. Vasoactive agent requirements were limited (Table 1). A complete set of haemodynamic data from the Starling™ monitor was available in all patients at all intra-operative time points. No side effect related to the use of NICOM was observed in any patient.

**Table 1:** Patient, pre-operative and intra-operative characteristics (*n* = 50)

Age (years)	56 ± 14
Sex (M/F)	33/17
BMI (kg m^−2^)	25.4 ± 5.3
ASA score	2 [2–2]
Left ventricular ejection fraction (%)	62 ± 9
Left ventricular ejection fraction < 40%	1 (2)
Left ventricular diastolic dysfunction	2 (4)
Right ventricular dysfunction	0
Comorbidities	
Ischemic heart disease	7 (14)
Valvular heart disease	0
Hypertension	22 (44)
Peripheral arterial disease	6 (12)
Diabetes mellitus	13 (26)
Chronic treatments	
Betablockers	10 (20)
Calcium channel inhibitors	11 (22)
Renin angiotensin system inhibitors	20 (40)
Type of surgery	
Video-assisted thoracic surgery - Lobectomy - Wedge resection	31 (62) 25 6
Open-chest surgery - Lobectomy - Wedge resection - Pneumectomy	19 (38) 15 3 1
Intra-operative data	
Duration of surgery (min)	150 ± 48
Duration of anaesthesia (min)	231 ± 65
Fluids requirements (ml)	1000 [862 to 1500]
Total hydric balance (ml)	822 ± 420
Bleeding (ml)	100 [50 to 238]
Vasoactive agents	
Ephedrine (mg)	9 [0 to 24]
Norepinephrine (μg kg^-1^ min^-1^)	0 [0 to 0.03]
Apgar surgical score^20^	7 [6 to 8]

Variables as reported as mean ± SD or median [IQR] or number (%).

**Fig. 1:**
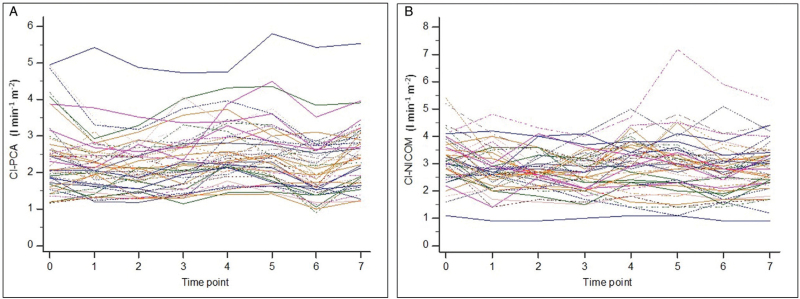
Individual values of cardiac index in patients performed by calibrated pulse contour analysis (a) and bioreactance (b) at eight intra-operative time points (*n* = 50). CI-PCA, cardiac index pulse contour analysis; CI-NICOM, cardiac index bioreactance.

There was a significant difference between CI-PCA and CI-NICOM: 2.4 ± 0.8 (range, 0.9 to 5.8) l min^-1^ m^−2^*vs.* 2.9 ± 0.9 (range, 0.9 to 7.2) l min^-1^ m^−2^, respectively (*P* < 0.001). Patients’ individual values of CI simultaneously recorded by both techniques at eight intra-operative time points are depicted in Fig. 1. The lowest and highest CI-PCA and CI-NICOM absolute values were significantly different (Table 2). Patients’ individual values of mean arterial pressure, heart rate and SVI recorded by both techniques at eight intra-operative time points are depicted in Annex 2. The lowest and highest SVI-PCA and SVI-NICOM absolute values were also significantly different (Table 2). Conversely, no significant difference was found in either maximum CI changes or in maximum SVI changes during the study period when performed with PCA and NICOM (Table 2). Of note, maximum percentage changes in both CI and SVI were clinically meaningful (>15%) and were not related to the measurement noise expected with the two techniques.

**Table 2:** Maximal intra-operative changes in haemodynamic variables

			Maximum difference	
			
Variable	Lowest value	Highest value	Absolute values	%
MAP (mmHg)	61 (59 to 64)	94 (91 to 101)	14 (−17 to 20)	19 (−20 to 29)
Heart rate (bpm)	59 (57 to 62)	85 (80 to 88)	14 (2 to 18)	21 (−3 to 27)
CI-PCA (l min^-1^ m^−2^)	1.78 (1.55 to 1.90)	2.68 (2.48 to 3.03)	−0.34 (−0.53 to 0.46)	−16 (−25 to 20)
CI-NICOM (l min^-1^ m^−2^)	2.20 (2.00 to 2.50)^b^	3.65 (3.52 to 3.93)^a^	−0.85 (−1.30 to −0.50)	−29 (−38 to −18)
SVI-PCA (ml m^−2^)	26 (23 to 29)	36 (34 to 42)	−7 (−10 to −4)	−20 (−27 to −13)
SVI-NICOM (ml m^−2^)	32 (29 to 35)^b^	53 (48 to 58)^a^	−14 (−16 to −12)	−32 (−38 to −25)

Values are median (95% CI). CI-PCA, cardiac index calibrated pulse contour analysis; CI-NICOM, cardiac index bioreactance; MAP, mean arterial pressure; SVI-PCA, stroke volume index calibrated pulse contour analysis; SVI-NICOM, stroke volume index bioreactance.

a CI-NICOM *vs.* CI-PCA and SVI-NICOM *vs.* SVI-PCA, *P* < 0.001. ^b^CI-NICOM *vs.* CI-PCA and SVI-NICOM *vs.* SVI-PCA, *P* < 0.01.

**Fig. 2:**
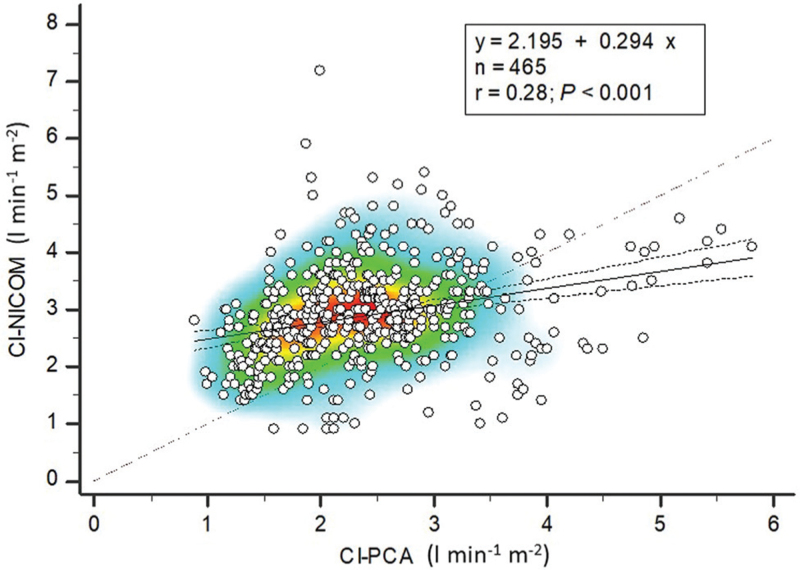
Heat maps showing the relationship between cardiac indexes performed by calibrated pulse contour analysis and bioreactance (*n* = 465 paired data points). Black dotted lines are 95% confidence interval. The reddish dotted line is the line of identity. CI-PCA, cardiac index pulse contour analysis; CI-NICOM, cardiac index bioreactance.

A weak but significant positive relationship was found between both techniques of CI assessment (Fig. 2). The heat map showing the best correlation was observed for CI values ranging from 2 to 3 l min^-1^ m^−2^. Comparing correlation coefficients between two-lung ventilation (T1) and one-lung ventilation (T2) with similar lateral positioning, we found no significant difference: 0.37 (95% CI, 0.07 to 0.61) *vs.* 0.46 (95% CI, 0.18 to 0.68), respectively (*P* = 0.639). Comparing correlation coefficients between closed-chest ventilation (T2) and open-chest ventilation (T3) with similar lateral positioning, we also found no significant difference: 0.46 (95% CI, 0.18 to 0.68) *vs.* 0.39 (95% CI, 0.09 to 0.63), respectively (*P* = 0.713). No difference was found between the correlation coefficients at the other pre-specified intra-operative time points. We conducted sensitivity analyses in the subgroup of video-assisted thoracic surgery only (*n* = 31 patients, 229 paired data points) and found a similar correlation between both methods: y = 1.289 + 0.356×; *r*^2^ = 0.111; *P* < 0.001.

**Fig. 3:**
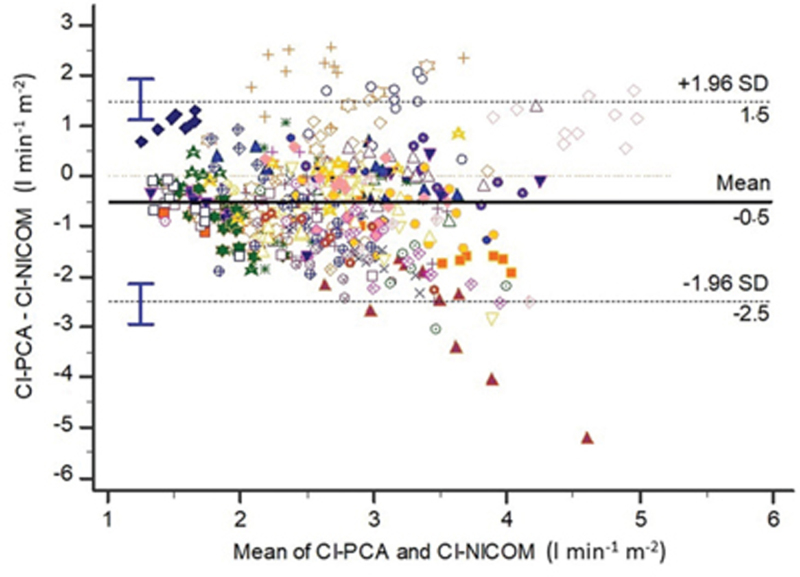
Bland–Altman analysis between cardiac indexes performed by calibrated pulse contour analysis and bioreactance (*n* = 465 paired data points). Symbols and colours refer to each patient (eight observations or more per individual). Blue bars are 95% CI of limits of agreement. CI-PCA, cardiac index pulse contour analysis; CI-NICOM, cardiac index bioreactance.

**Fig. 4:**
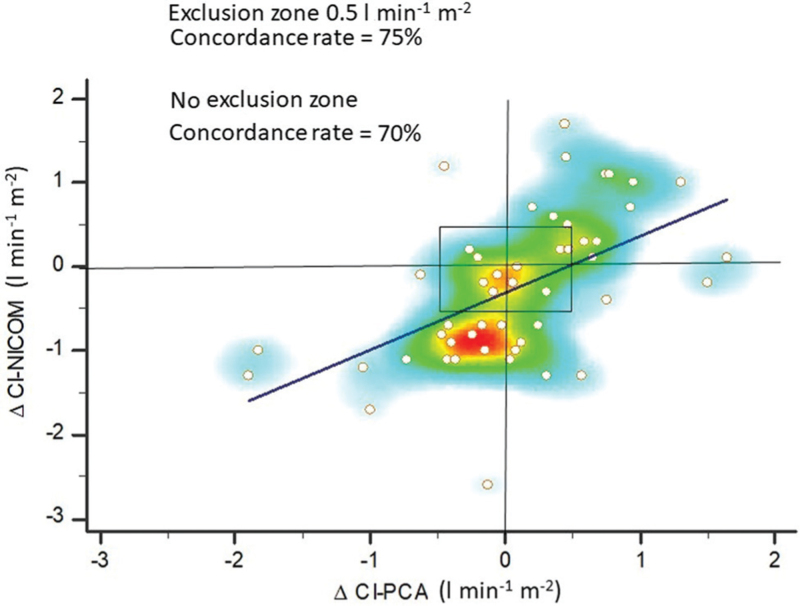
Four-quadrant plot with heat map showing the concordance between maximum differences in cardiac indexes performed by calibrated pulse contour analysis and bioreactance (*n* = 50 paired data points). The exclusion zone (blue triangle) was set to be 0.5 l min^−1^ m^−2^. The dark blue line is the linear regression showing the relationship between the maximum changes in CI-PCA and CI-NICOM. Concordance rates are shown with and without data exclusion. ΔCI-PCA, maximum difference cardiac index pulse contour analysis; ΔCI-NICOM, maximum difference cardiac index bioreactance.

Bland–Altman analysis with multiple measurements per individual is depicted in Fig. 3. Taking CI-PCA as the reference method, there was a systematic overestimation by CI-NICOM of 21% (0.5 l min^-1^ m^−2^), while the lower and upper limits of agreement were large: -2.49 and 1.47 l min^-1^ m^−2^, respectively (Fig. 3). The overall percentage error was 77%. Percentage errors during two-lung ventilation (T1) and one-lung ventilation (T2) were 78 and 69%, respectively (*P* = 0.365). Percentage errors during closed-chest ventilation (T2) and open-chest ventilation (T3) were 69 and 73%, respectively (*P* = 0.695). There was a significant moderate positive relationship between maximum changes in CI over time when measured by both techniques: y = -0.307 + 0.676×, *r*^2^ = 0.29, *P* < 0.001 (Fig. 4). The heat map showed that the best correlation was observed for changes below 1.0 l min^-1^ m^−2^. The concordance rate was 75 and 70% with and without an exclusion zone set to be 0.5 l min^−1^ m^−2^, respectively (Figure 4).

## Discussion

The main results of the present study are that in a specific cohort of intermediate-risk patients undergoing thoracic surgery for cancer, the use of chest bioreactance to continuously and non-invasively monitor cardiac output and stroke volume is well tolerated and easily feasible in all patients at all intra-operative time points and shows significant expected spontaneous variations; and is moderately correlated with and provides moderate trending ability with, but is not interchangeable with calibrated PCA used as the reference method over a wide range of CI values.

Despite a convincing body of literature^1–3^ and recent experts’ recommendations^4^ suggesting that advanced haemodynamic monitoring is mandatory to enable peri-operative GDFT, monitoring cardiac output in addition to arterial pressure remains uncommon in routine clinical practice. One explanation is that the vast majority of careproviders believe that cardiac output monitoring by means of reference methods is too invasive and complicated, and that cardiac output monitoring by means of non-invasive, operator-independent and easy-to-use methods is not reliable and not interchangeable with thermodilution.^6,7,21^ Thoracic surgery patients undergoing one-lung ventilation and open-chest surgery are challenging regarding GDFT, but an individualised strategy seems superior to ‘standard of care’.^15^ Despite this, invasive reference methods of cardiac output monitoring are not routinely used and validation of non-invasive tools have produced contradictory results.^14,15^ In the current study, we found that chest bioreactance was easily feasible in all patients at all intra-operative time points and provided spontaneous, significant and physiologically plausible variations whose magnitude was beyond the noise of measurement. The present finding is of interest, as it appears that thoracic surgery and one-lung ventilation do not impair the exploration of frequency spectra variations of delivered oscillating current across the thorax. Our results are in accordance with previous studies evaluating the ability of bioreactance in other kinds of surgery and other types of patients.^12,13,22,23^ However, the comparison of both absolute values and maximum changes of CI-NICOM with CI-PCA (initially calibrated by echocardiography and used as the reference method) only showed a weak to moderate positive relationship, the best correlation being observed within the range of ‘normal’ CI values (illustrated by heat map imaging). Furthermore, bioreactance systematically overestimated CI by nearly 20%, while limits of agreement were large and the percentage error was far beyond the usual threshold of 30%: this means that the two methods are not interchangeable, as already reported in colorectal surgery patients.^13^ Interestingly, both correlation coefficients and percentage errors were similar whatever the ventilation mode (two-lung and one-lung ventilation) or the open/closed chest condition, at least suggesting that bioreactance is robust. As regards the concordance rate, with or without an exclusion zone, the trending ability was only moderate, indicating that CI-NICOM and CI-PCA could vary in opposite directions. This last point is crucial and deserves further investigation, as it could compromise the ability of bioreactance to guide GDFT in that specific surgical setting.

Several limitations of the present study must be highlighted. Firstly, in the strictest sense, invasive radial arterial PCA, even initially calibrated by means of echocardiography, cannot be considered as a reference method, so that we cannot say definitively which technique overestimated or underestimated the true value of CI. PCA is however a validated method to conduct GDFT and improve patients ‘outcome in non-cardiac surgery (including thoracic surgery),^9^ able to detect rapid and small changes in CI and SVI,^24^ even smaller than those we report in the current study. The majority of the currently available literature suggests PCA or oesophageal Doppler as the most desirable choices to guide fluid administration.^25^ Surveys show that CI-PCA is the most broadly used method in moderate-to-high risk surgical patients, even though we do not use it systematically in our thoracic surgery patients consequently, we cannot exclude a selection bias in our convenience series. Oesophageal Doppler is more difficult to use at the bedside,^13^ and despite our initial intention to use it for the whole cohort of patients, we quickly abandoned it following the first ten patients. The calibrated ProAQT/PulsioFlex system has been found to reliably track changes in cardiac output after volume expansion or pharmacological interventions.^26^ The purpose of calibration is to correct the estimate of the constant *k* for changes in afterload,^27^ and the majority of clinical data suggests that calibration improves accuracy: that is the reason why we chose to use it in the current study. We did not consider using stroke volume variation or pulse pressure variation, as both have been shown as useless for predicting fluid responsiveness in thoracic surgery.^14^ Secondly, a single simultaneous measurement was performed at each time point: this measurement actually represents the mean values of stroke volume and CI over the specific moving average periods of the monitors. Thirdly, our results do not mean that bioreactance is not useful for guiding GDFT in thoracic cancer surgery: the present study was not designed to assess the ability of the techniques to decrease postoperative morbidity and/or mortality but only to compare them. Moreover, we assessed trending ability to detect maximum changes in CI-NICOM among pre-specified time points and not specifically in response to fluid challenge or vasoactive agents. Future studies should address that crucial point before making any recommendations on the use of bioreactance for routine GDFT in clinical practice. Shorter durations of ventilation and hospital-stay have been reported in surviving trauma patients with complex injuries and valvular heart surgery patients when bioreactance was used.^23,28^ Fourthly, despite the apparently successful use of concordance analysis in several studies,^29^ CI-NICOM has its weaknesses, even when using exclusion zones to reduce the noise from small changes in cardiac output.^30^ Furthermore, the distribution of spread of CI changes will also affect the concordance rate. In previous studies in which agreement was poor according to the more than 30% criteria, the concordance rate ranged from 67 to 88% when exclusion of central data (0.5 to 1.0 l min^-1^ or 15%) was applied,^19^ meaning our results were somewhat expected. More sophisticated statistical methods such as trend interchangeability could be used to further assess the trend ability of chest bioreactance.^31,32^

In conclusion, bioreactance is a feasible and well tolerated technique to continuously and easily monitor cardiac output non-invasively in thoracic cancer surgery patients. Intra-operatively, it is able to detect significant spontaneous and pharmacologically induced changes over time. A moderate correlation, wide limits of agreement and a moderate concordance with calibrated PCA indicate a need for further studies before making any recommending as to its wider use in routine practice, especially when the aim is to guide individualised GDFT in order to improve patient outcomes.

## Supplementary Material

Supplemental Digital Content

## Supplementary Material

Supplemental Digital Content
