# Supplementary material for: Non-invasive continuous cardiac output monitoring in thoracic cancer surgery: A comparative study between calibrated pulse contour analysis and chest bioreactance
Source: Eur J Anaesthesiol Intensive Care. 2022 Sep 15;1(4):e006. doi: 10.1097/EA9.0000000000000006 (PMC11783632; doi:10.1097/EA9.0000000000000006)

**Annex 2:** Patients individual values of mean arterial pressure (A), heart rate (B), stroke volume performed by calibrated pulse contour analysis (C) and bioreactance (D) at 8 intraoperative time points (n = 50).

HR: heart rate; MAP: mean arterial pressure; SVI-PCA: stroke volume index pulse contour analysis; SVI-NICOM: stroke volume index bioreactance.


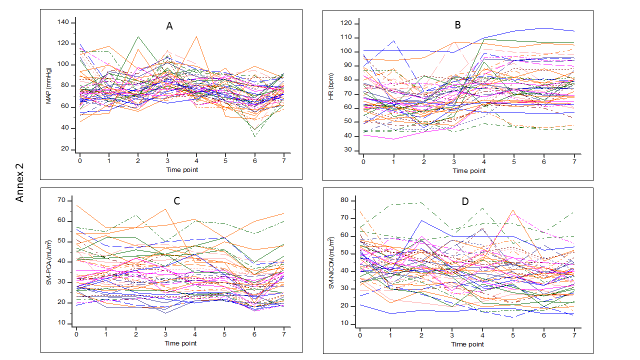

Supplement: Supplemental Digital Content [file ejaic-1-e006-s002.docx]
